# Key role of 15-LO/15-HETE in angiogenesis and functional recovery in later stages of post-stroke mice

**DOI:** 10.1038/srep46698

**Published:** 2017-04-24

**Authors:** Di Wang, Yu Liu, Li Chen, Pengyan Li, Youyang Qu, Yanmei Zhu, Yulan Zhu

**Affiliations:** 1 Department of Neurology, The Second Affiliated Hospital of Harbin Medical University, 246 Xuefu Road, Harbin, Heilongjiang 150086, China

## Abstract

This study sought to clarify the effects of 15-lipoxygenase/15-hydroxyeicosatetraenoic acid in angiogenesis and neurological functional recovery after cerebral ischaemic stroke in mice. *In vivo*, we performed behavioural tests to determine functional recovery after stroke. Double immunofluorescence staining of CD31 and Ki67/PCNA was performed to evaluate the effects of 15-lipoxygenase/15-hydroxyeicosatetraenoic acid on angiogenesis in an MCAO mouse model. *In vitro*, we investigated the effects of 15-hydroxyeicosatetraenoic acid on BMVEC proliferation and migration. Our results show that MCAO upregulates 15-lipoxygenase expression in a time-dependent manner, especially in later stages of post-stroke. We confirmed that cerebral infarct area was reduced and neurological dysfunction was gradually attenuated after stroke, while 12/15-lipoxygenase knockout mice exhibited the opposite effects. Furthermore, immunofluorescence studies revealed 15-lipoxygenase increased the proliferation of mouse brain vascular endothelial cells in a time-dependent manner, while 12/15-lipoxygenase knockout blocked these effects. Moreover, 15-hydroxyeicosatetraenoic acid promoted proliferation and tube formation in BMVECs. These results demonstrate positive influence of 15-lipoxygenase/15-hydroxyeicosatetraenoic acid in angiogenesis and neuronal recovery after ischaemic stroke in mice. We also confirmed the PI3K/Akt signalling pathway was necessary for the effects of 15-hydroxyeicosatetraenoic acid in regulation of BMVEC proliferation and migration, which may potentially be a novel target for the recovery from ischaemic stroke.

Stroke is an acute cerebrovascular disorder caused by abnormal blood supply to the brain due to ischaemia or haemorrhage, resulting in long-term neurological impairment among patients^1^,^2^. Therapeutic options for clinical management remain quite limited. Several studies have demonstrated that stimulation of angiogenesis generates new vessels, increases collateral circulation and may contribute to improvement in long-term neurological function after stroke^3^,^4^,^5^.

Angiogenesis is characterized by sprouting of endothelial cells (ECs) from pre-existing blood vessels and involves migration, proliferation, and differentiation of ECs^6^,^7^,^8^,^9^,^10^. Excessive proliferation, migration, adhesion and tube formation of ECs have been demonstrated to promote new vessel growth and angiogenesis^11^,^12^. Although the contribution of endothelium has been studied over past decades, the cellular and molecular mechanisms underlying post-stroke angiogenesis have not yet been fully described.

Our group has previously reported that hypoxia upregulates the expression of 15-lipoxygenase (15-LO), which catalyses arachidonic acid (AA) into 15-hydroxyeicosatetrienoic acid (15-HETE) in the brain, and that 15-LO/15-HETE plays an important role in brain vascular contractile reactivity following ischaemia^13^,^14^. Numerous studies have shown that 15-LO/15-HETE axis is linked to regulation of both embryonic and pathologic angiogenesis in different tissues^5^,^6^,^7^,^8^,^9^,^15^,^16^,^17^. A recent report confirmed that 15-HETE participates in hypoxia-induced pulmonary artery angiogenesis^18^. However, the function and mechanisms of 15-LO/15-HETE in ischaemic stroke, which are related to angiogenesis, are unclear.

In this study, we evaluated the expression and localization of 15-LO in MCAO brains of wild-type (WT) and 12/15-LO^−/−^ mice and in brain microvascular endothelial cells (BMVECs). We performed behavioural tests to determine functional recovery after stroke; then, double immunofluorescence staining of CD31 and Ki67/PCNA was applied on brain tissue to evaluate the effects of 15-LO/15-HETE on angiogenesis in an MCAO mouse model. We also determined the role of 15-HETE in regulation of cell proliferation, along with effects on cell migration.

## Results

### The expression of 15-LO is increased after MCAO in brain vasculature

**Figure 1:**
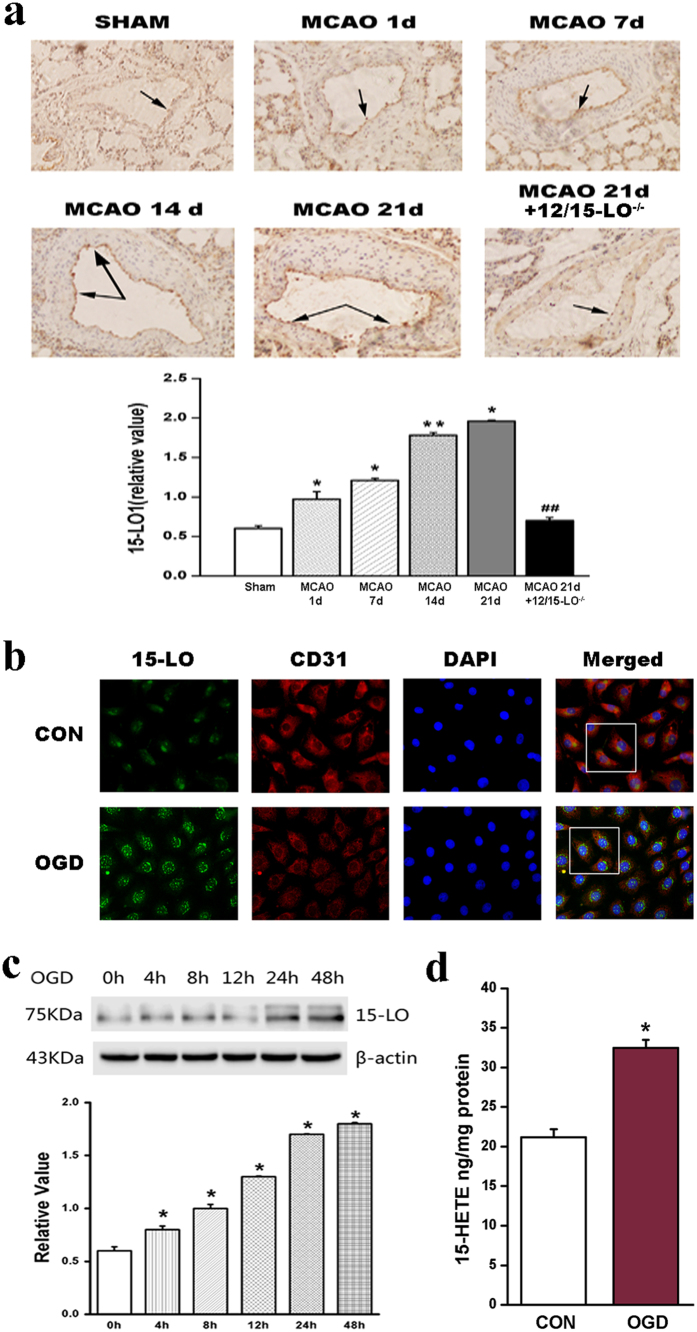
15-lipoxygenase (15-LO) expression is increased in brain artery endothelium of post-stroke mice and OGD-induced BMVECs. (**a**) Immunohistochemical staining of 15-LO in brain arteries from WT mice suffered MCAO for different days and 12/15-LO^−/−^ mice (n = 10, *p < 0.05, **p < 0.01, ^##^*p* < 0.01). Values are represented as the mean ± S.E.M. (**b**) BMVECs were fixed and stained with anti-15-LO (green), anti-CD31 (red) and DAPI to stain nuclei (blue). Merged images show 15-LO colocalizes to CD31 (a marker of ECs). Scale bars are 25 μm. Images shown are representative of at least three independent experiments. (**c**) Quantification of 15-LO protein levels in BMVECs under OGD for different time points (0, 4, 8, 12, 24, 48 hours; n = 4, *p < 0.05). Values are represented as the mean ± S.E.M. (**d**) The endogenous level of 15-HETE was measured by15-HETE EIA kit in BMVECs. OGD increases the endogenous15-HETE production compared with normoxic conditions (n = 4, *p < 0.05).

To examine the expression and distribution of 15-LO in brain vasculature, an MCAO mouse model was constructed. We localized 15-LO in the brain using immunohistochemistry on tissue sections. The expression of 15-LO was upregulated after MCAO in a time-dependent manner, which was mainly localized in vascular intima (Fig. 1a) from the 1st day to the 21st day after MCAO treatment, and the level of 15-LO was most obvious in the 21st day (later stages of post-stroke) after MCAO. 12/15-LO is the murine orthologue of human 15-LO; therefore, to clarify the role of 15-LO in mice, we used 12/15-LO knockout (12/15-LO^−/−^) mice in the following experiments. As we observed, the expression of 15-LO decreased to a lower level in 12/15-LO^−/−^ mice (21st day after MCAO), accompanied by a thinning of blood vessels (Fig. 1a). Our results confirmed that MCAO increases the expression of 15-LO in the brain vasculature, and this effect was intense during the late period of post-stroke. One animal from the 12/15-LO^−/−^ group was excluded due to subarachnoid haemorrhage. No mortality was seen in either group.

### Expression and subcellular distribution of 15-LO/15-HETE under OGD in cultured BMVECs

Oxygen-glucose deprivation (OGD) is a model of ischaemia and hypoxia *in vitro* we used to ascertain whether OGD regulates 15-LO expression *in vitro*. We examined the expression of 15-LO protein using immunofluorescent staining with a 15-LO antibody. The findings in Fig. 1b show that most elevated 15-LO expression colocalized with CD31 (a marker of ECs), and mainly distributed in nuclei of the OGD group. This suggests that ischaemia and hypoxia upregulate the expression of 15-LO *in vitro*, and 15-LO distributes mainly in endothelium. In addition, 15-LO protein expression under OGD was further investigated using western blot analysis (Fig. 1c). The results showed an increase of 15-LO, which peaked at 48 h after OGD.

To assess whether OGD has a promoting effect on endogenous 15-HETE generation, we examined the 15-HETE level in BMVECs using the15(S)-HETE EIA Kit. Our results showed that OGD promotes the formation of endogenous 15-HETE (Fig. 1d). These data confirmed that OGD induces an increase of 15-LO/15-HETE in brain endothelium.

### 15-LO/15-HETE protects against ischaemic brain infarction and improves neurological function

**Figure 2:**
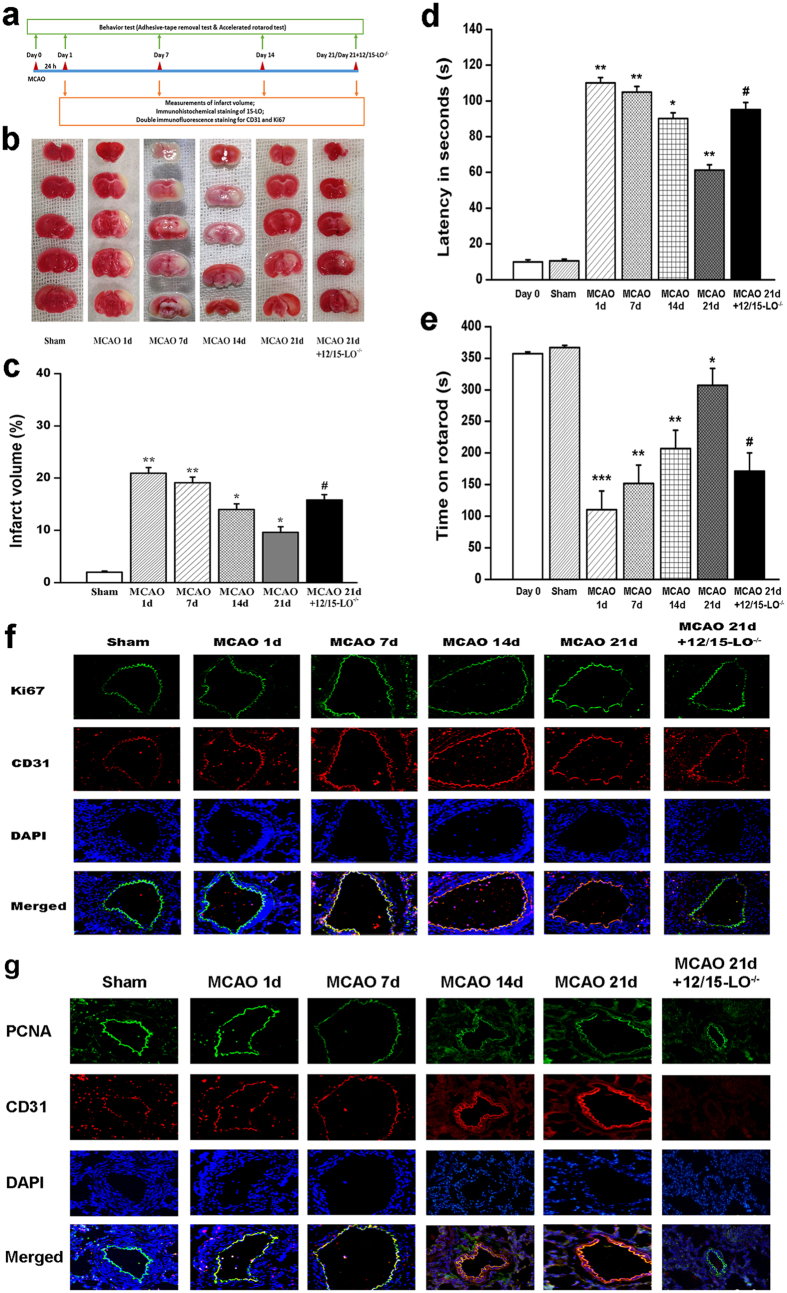
15-lipoxygenase (15-LO) protects against ischaemic brain infarction, improves neurological function and increases the proliferation of brain vascular endothelial cells in post-stroke mice. (**a**) Experimental design of the present study. Ischaemic stroke was induced by MCAO, behavioural tests and other experiments were performed at the indicated intervals. (**b**) Representative TTC-stained brain sections from sham-operated control mice and different post-stroke time point mice. (**c**) Quantification of the infarct volume shown in B (n = 10, *p < 0.05, **p < 0.01, ^#^*p* < 0.05). Values are represented as the mean ± S.E.M. (**d**) Adhesive-tape removal test performed at different times after reperfusion in WT mice and 12/15-LO^−/−^ mice. (**e**) Rotarod test performed at different times after reperfusion in WT mice and 12/15-LO^−/−^ mice. (n = 10, *p < 0.05, **p < 0.01, ***p < 0.001, ^#^*p* < 0.05). Values are represented as the mean ± S.E.M. (**f**) (**g**) Blood vessels in the MCAO adductor brain tissues from WT and 12/15-LO^−/−^ mice were analysed by double immunofluorescence staining for anti-Ki67 (green)/anti-PCNA (green), anti-CD31 (red) and DAPI to stain nuclei (blue). Merged images show Ki67/PCNA co-localizes to brain vascular endothelial cells. Co-localization was most apparent 21 days after stroke, and 12/15-LOKO blocked these effects. Scale bars are 50 μm. Images shown are representative of at least three independent experiments.

To evaluate the protective effects of 15-LO/15-HETE against stroke, we measured the infarct area in a mouse model of focal ischaemia. There were no significant differences in the consumption of food and drinking solution between different groups. At 24 h, 7 d, 14 d, 21 d post-stroke, animals were killed, and infarcts were evaluated with TTC staining (Fig. 2b). Infarct analysis revealed that after 60 min MCAO, there was no detectable infarction in the sham-operated control group. WT mice survival for 1 d had a total infarct volume of 21.03 ± 2.3% (n = 10), WT mice survival for 7 d had a total infarct volume of 18.5 ± 1.8% (n = 10), WT mice survival for 14 d had a total infarct volume of 15.5 ± 2.5% (n = 10), and WT mice survival for 21 d had a total infarct volume of 9.8 ± 1.9% (n = 10). In contrast, 12/15-LO^−/−^ mice survival for 21 d had a total infarct area of 16.9 ± 2.3% (n = 10, *p* < 0.05) (Fig. 2c). These results illustrated that the infarct volume was gradually decreased after stroke and correlated with increased 15-LO expression.

Table 1 shows neurological deficit scores (NDS) after 60 min of MCAO and 24 h of reperfusion in different experimental groups. NDS was significantly higher in mice surviving for the 1 d group compared with the sham control group (*p* < 0.05). All MCAO mice exhibited progressive recovery during the experimental days (1, 7, 14, and 21), and NDS gradually decreased. Compared with the 21-day survival group, NDS was increased in 12/15-LO^−/−^ mice (n = 10, *p* < 0.05). These data showed that neurological function was gradually ameliorated after stroke along with decreased infarct area and increased 15-LO.

Neurological function was assessed using Bederson scores. All MCAO WT mice exhibited progressive recovery during the experimental days (1, 7, 14, 21), and NDS was gradually decreased. Compared with WT mice survival for 21 d, NDS was increased in 12/15-LO^−/−^ mice. (n = 10 per group, *p < 0.05; ^#^*p* < 0.05; values are denoted as the means ± SEM).

### 15-LO/15-HETE reduces latency of adhesive-tape removal and improves the performance in rotarod assay

Functional deficits are common neurological sequelae in patients with brain injuries induced by stroke and in animal models of stroke. The effects of 15-LO/15-HETE on neurological function were studied using behavioural tests (n = 10 per experimental group). Pre-trained animals were tested for latency in the adhesive-tape removal test, which is a measure of somatosensory dysfunction after cerebral ischaemia in mice^19^. Figure 2d shows that stroke significantly increased tape removal latency and that this latency was significantly improved by 15-LO/15-HETE.

The positive effects of 15-LO/15-HETE on motor outcomes were also observed the accelerated rotarod test (Fig. 2e). There were no significant differences in performance between pre-ischaemia groups and the sham control group (*p* > 0.5). From days +1 to +14 after MCAO, mice showed strong decreases in their performances compared to the corresponding pre-ischaemia and sham groups (*p* < 0.01). WT mice survival for 21 d showed a significant improvement of their performances on the rotarod compared with the sham group (Fig. 2e, *p* < 0.05). 12/15-LO^−/−^ mice tended to perform worse compared to WT mice surviving for 21 d (Fig. 2e, *p* < 0.05), suggesting that 15-LO/15-HETE improved performance in post-stroke rotarod impairment tests.

### 15-LO/15-HETE increases the proliferation of brain vascular ECs in post-stroke mice

Next, we investigated whether 15-LO/15-HETE regulated the angiogenesis of brain vasculature after MCAO. As shown in Fig. 2f, along with the time after stroke, immunofluorescence showed that enhanced Ki67 (marker of proliferation) colocalized with CD31 (marker of ECs) was most apparent on the 21st day after stroke, and 12/15-LO gene knockout blocked these effects. We also assessed PCNA (proliferating cell nuclear antigen), which is recognized as an angiogenic marker in many studies^20^. The results showed that enhanced PCNA colocalized with CD31 was most apparent 21 days after stroke, and 12/15-LO gene knockout blocked these effects (Fig. 2g). These data suggest that 15-LO/15-HETE promotes brain vascular endothelium proliferation after ischaemic injury, which confirms brain angiogenesis.

### 15-HETE induces BMVEC proliferation and migration under OGD

**Figure 3:**
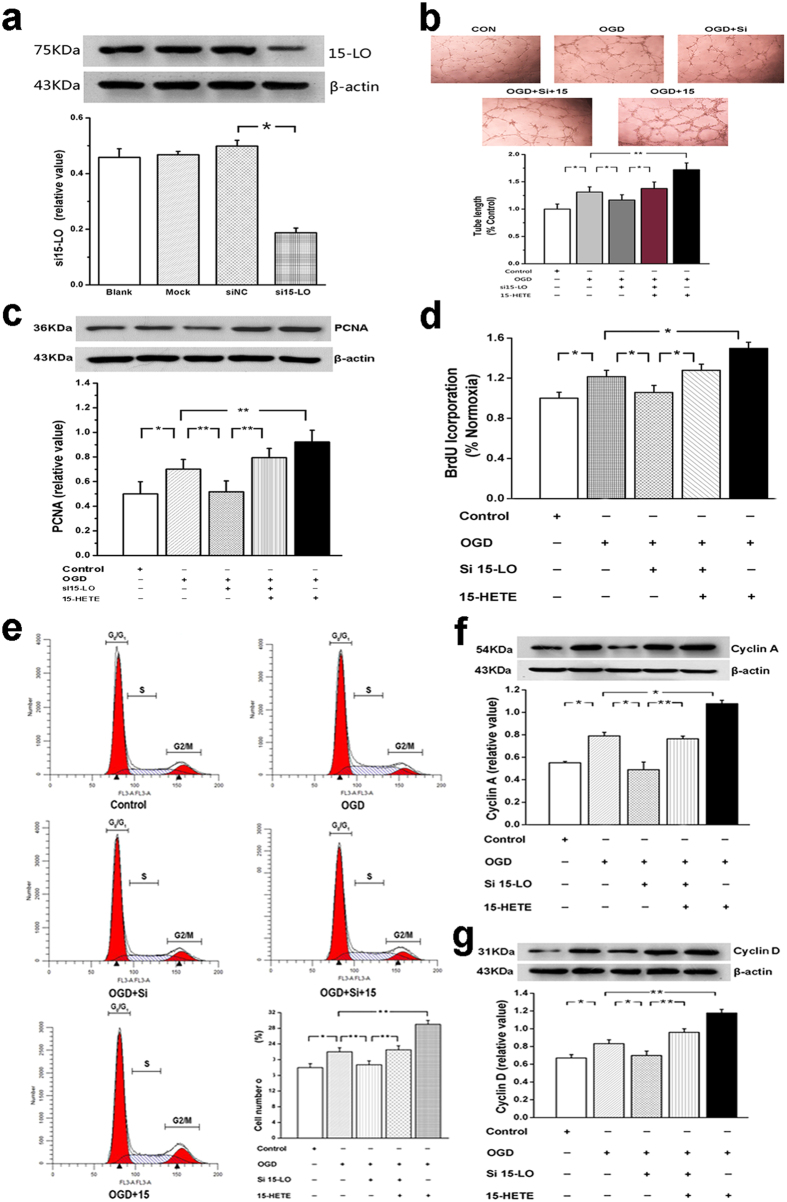
15-Hydroxyeicosatetraenoic acid (15-HETE) induces brain microvascular endothelial cell (BMVEC) proliferation and migration after OGD and induces OGD effects and the 15-LO/15-HETE axis on cell cycle in BMVECs. (**a**) 15-LO protein expression was down-regulated after transfecting the 15-LO siRNA sequence into BMVECs. (**b**) BMVECs were subjected to the tube formulation assay. 15-LO siRNA inhibited cell tube formation induced by OGD, which was attenuated by exogenous 15-HETE (1 μmol/mL). (**c**) OGD and exogenous 15-HETE upregulated proliferating cell nuclear antigen (PCNA). (**d**) 15-LO siRNA decreased 5-bromodeoxyuridine (BrdU) incorporation compared with the OGD group; however, the incorporation was significantly enhanced by both endogenous and exogenous 15-HETE. (n = 4, *p < 0.05, **p < 0.01). Values are represented as the mean ± S.E.M. (**e**) Cells accumulated in S and G_2_/M under OGD. This was reversed by 15-LO inhibition. (**f**) OGD increased cyclin An expression and this was reversed by siRNA for 15-LO. (**g**) Expression of cyclin D in BMVECs was similarly affected by OGD or 15-HETE. (n = 4, *p < 0.05, **p < 0.01). Values are represented as the mean ± S.E.M.

To determine whether 15-HETE, the 15-LO metabolite of arachidonic acid, is angiogenic, we performed the BMVEC tube formation assay. First, we used specific siRNA to silence 15-LO gene expression in BMVECs, confirming with western blot analysis that 15-LO was adequately knocked down (Fig. 3a). Our results showed that OGD stimulated BMVECs tube formation compared with control in normoxia (Fig. 3b). After silencing 15-LO gene expression in BMVECs, which results in decreased endogenous 15-HETE, BMVEC tube formation was inhibited, while tube formation was increased by adding exogenous 15-HETE (Fig. 3b). Our results showed that both exogenous 15-HETE and endogenous 15-HETE generated in OGD-stimulated BMVEC migration. We also analysed the expression of proliferating cell nuclear antigen (PCNA) in BMVECs. We found that OGD increased the expression of PCNA, and the effect was decreased in the presence of siRNA. Exogenous 15-HETE upregulated the level of PCNA (Fig. 3c). To assess the population of cells which are actively synthesizing DNA, 5-bromodeoxyuridine incorporation was measured. Our results showed that treatment with 15-LO siRNA markedly inhibited cell proliferation, whereas exogenous 15-HETE significantly enhanced 5-bromodeoxyuridine incorporation (Fig. 3d). These results showed that 15-LO/15-HETE may be involved in mediating proliferation and migration of BMVECs *in vitro*.

### 15-HETE promotes cell-cycle progression and regulates expression of cell-cycle-related proteins

To understand whether OGD affects cell cycle progression through the 15-LO/15-HETE pathway, the number of cells in different cell cycle phases was measured by flow cytometry. The results show that endogenous 15-HETE increased the percentage of cells in the G_2_/M + S phase. Treatment with 15-LO siRNA suppressed cell cycle progression and more BMVECs arrested at the G_0_/G_1_ phase. In contrast, the accumulation of the G_2_/M + S phase was increased in presence of exogenous 15-HETE (Fig. 3e). A-type cyclins play an important role in the stimulation of cyclin-dependent kinases and thus regulate the S to G_2_/M phase transitions. Cyclin D is known to regulate the transition of cell cycle from G_1_ to S phase^21^,^22^; thus, we analysed the expression of cyclin A and cyclin D in BMVECs. OGD increased the expression of cyclin A and cyclin D, and these effects were attenuated after treatment with the siRNA for 15-LO (Fig. 3f,g). These results suggested that 15-HETE has an impact on the cell cycle activity and induces BMVEC proliferation, eventually contributing to brain vascular angiogenesis.

### 15-HETE increases cell proliferation and migration in a PI3K/Akt-dependent manner

**Figure 4:**
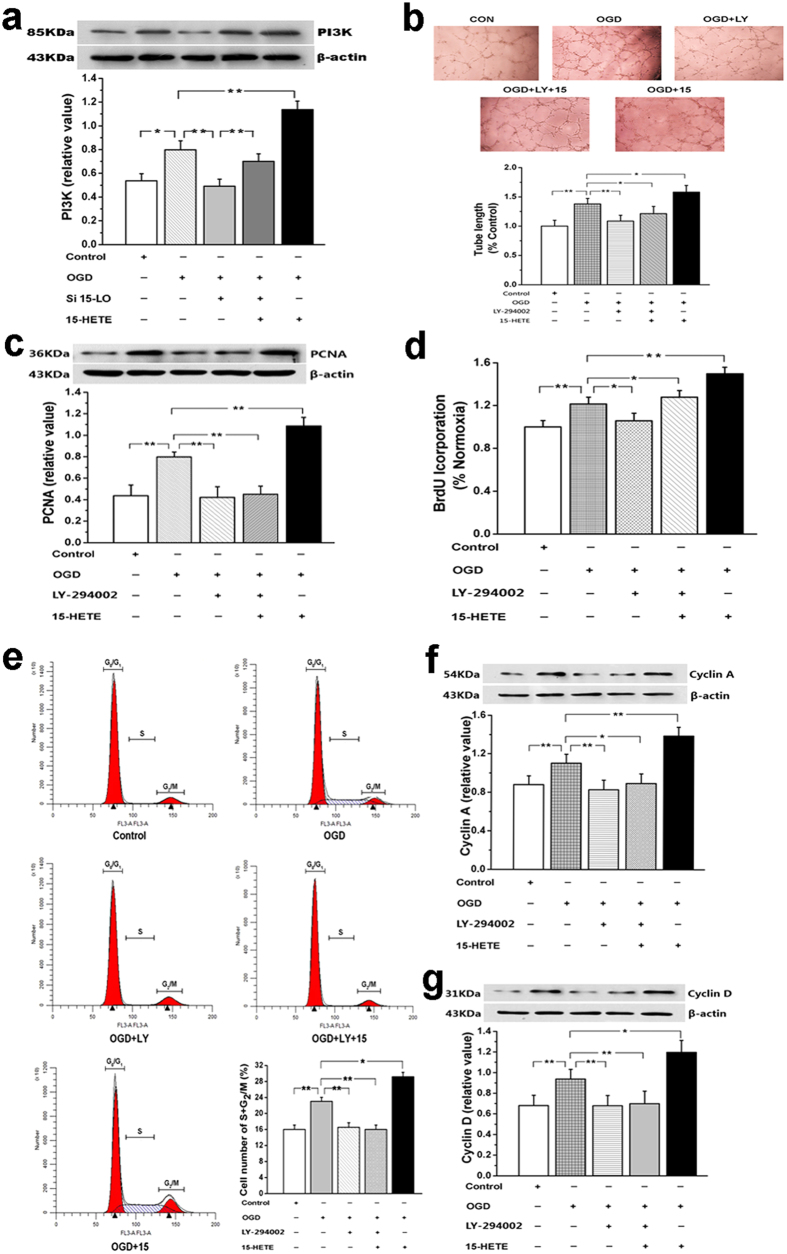
15-HETE increases proliferation and migration of BMVECs in a PI3K/Akt-dependent manner. (**a**) In BMVECs under OGD conditions, both endogenous and exogenous 15-HETE (1μmol/L) increased PI3K/Akt expression. (n = 4, *p < 0.05, **p < 0.01). Values are represented as the mean ± S.E.M. (**b**) Pretreatment with LY-294002 blocked the effects of 15-HETE on cell migration. (**c**) The increased PCNA expression after 15-HETE under OGD was blocked by the PI3K/Akt inhibitor. (**d**) Pretreatment of LY-294002 blocked the exogenous 15-HETE-induced BrdU incorporation. (n = 4, *p < 0.05, **p < 0.01). Values are represented as the mean ± S.E.M. (**e**) Flow cytometry for cell-cycle analysis indicated that 15-HETE stimulated BMVEC progression into G_2_/M + S phase, and this effect was blocked by LY-294002. (**f**) LY-294002 also blocked the increase in cyclin A or (**g**) cyclin D, induced by 15-HETE. (n = 4, *p < 0.05, **p < 0.01). Values are represented as the mean ± S.E.M.

To determine whether PI3K/Akt signalling was altered along with BMVEC proliferation and migration, we measured levels of PI3K in BMVECs. Our results showed that OGD upregulates the expression of PI3K, which was inhibited by treatment with the siRNA for 15-LO. This expression was higher in the presence of exogenous 15-HETE (Fig. 4a). Then, we blocked the PI3K/Akt pathway with LY-294002. Tube formulation assays showed that the group where the PI3K/Akt pathway was blocked with LY-294002 produced a significant inhibition of the migratory capability of BMVECs induced by both endogenous and exogenous 15-HETE (Fig. 4b). Moreover, the expression of PCNA induced by 15-HETE was also abrogated by the PI3K/Akt inhibitor (Fig. 4c). Figure 4d demonstrated that increased effects of endogenous 15-HETE on cell 5-bromodeoxyuridine incorporation was significantly suppressed by LY-294002. These data suggest that the effect of 15-HETE on BMVEC migration and proliferation was mediated by the PI3K/Akt pathway.

### Role of PI3K/Akt signalling in 15-HETE-induced cell-cycle progression

We next assessed the involvement of the PI3K/Akt pathway in the effects of 15-HETE on cell cycle. Treatment of BMVECs with LY-294002 increased the fraction of cells in the first peak (G_0_/G_1_) and correspondingly decreased the fraction of cells in the S and G_2_/M phases. In the presence of LY-294002, 15-HETE had no effect on the cell-cycle profile (Fig. 4e). As shown in Fig. 4f and g, cyclin A and cyclin D expression were substantially increased after OGD but, in the LY-294002-treated cells, these cyclins were not increased by OGD plus 15-HETE. These results confirm an important role of the PI3K/Akt pathway in cell circle progression under OGD.

## Discussion

The experimental evidence from this study clearly demonstrates that the elevation of 15-LO in brain vascular endothelium triggered by ischaemia and hypoxia is time dependent, and the level of 15-LO was most obvious on the 21st day (later stages of post-stroke) after MCAO. Our study also clarified the pivotal role of 15-LO/15-HETE in post-stroke behavioural recovery and post-stroke angiogenesis. The expression of 15-LO/15-HETE improved functional recovery, as measured by the adhesive-tape removal test and the accelerated rotarod test, which are sensitive indexes for assessing motor impairment after focal ischaemia and traumatic brain injury^23^,^24^. We also found that infarct volume gradually decreases after stroke along with the expression of 15-LO. We observed that 15-LO significantly enhanced angiogenesis, confirmed by the increased fluorescence intensity of proliferous ECs in functional blood vessels and Ki67/PCNA co-labelling (Fig. 2). This suggests that 15-LO/15-HETE enhances functional recovery in parallel to the induction of angiogenesis. Moreover, we demonstrated that 15-HETE, a product of 15-LO, stimulated proliferation and migration of BMVECs, regulated expression of cell cycle-related proteins, and modulated the cell cycle *in vitro*. Furthermore, the PI3K/Akt signalling pathway was involved in the proliferation and migration as well as regulation of cell cycle in BMVECs induced by 15-HETE. This study identified, for the first time, that ischaemia and hypoxia affects 15-LO expression in the brain vasculature, 15-LO/15-HETE promotes angiogenesis and neuronal recovery in late period of post-stroke mice and that 15-HETE stimulates proliferation and migration of BMVECs, via PI3K/Akt signalling.

In this study, an MCAO model using C57BL/6 WT mice and 12/15-LO^−/−^ mice was established to reveal the protective effect of 15-LO/15-HETE on cerebral ischaemic injury. This ischaemic injury model mimics a clinical MCAO, the most prevalent form of human stroke^25^. A blockage in the vessel leads to obstruction of collateral blood supply and produces focal ischaemic lesions in the striatum and motor cortex^26^,^27^. 12/15-LO is the murine orthologue of human 15-LO. Therefore, to clarify the role of 15-LO in mice, we used 12/15-LO knockout (12/15-LO^−/−^) mice in our experiments. Severe reductions in blood flow to brain tissue results in a lack of oxygen and nutrient transportation, leading to brain hypoxia. Hypoxia is known to induce the expression of 15-LO and 15-HETE formation in many different tissues, which plays an important role in vascular angiogenesis^22^,^27^. Thus, we considered whether 15-LO/15-HETE would be affected during MCAO-induced angiogenesis. We found a time- dependent augmentation of 15-LO expression in vascular endothelium following MCAO and, in cultured BMVECs, which declined in 12/15-LO^−/−^ mice. In our model, the time-dependent increase in infarct volume peaked after 24 h of reperfusion and was inversely proportional to the level of 15-LO expression. As 15-LO gradually increased following insult, the infarct volume decreased, and neurological function gradually improved after stroke (Fig. 2); all these effects were reversed by 12/15-LOKO. Our results suggest that 15-LO expression is associated with functional improvement after ischaemic stroke.

During and after stroke, brain vasculature becomes leaky and unstable, and the normally impermeable BBB breaks down. There is increasing evidence that angiogenesis, through new blood vessel formation, results in improved collateral circulation and may impact the long-term recovery of patients^15^,^28^,^29^. Newly formed vessels would allow increased blood flow, thus increasing perfusion of the damaged ischaemic area. Studies using human brain samples suggest that active angiogenesis occurs 3–4 days after stroke and indicates that the number of new vessels in ischaemic penumbral regions are correlated with survival^30^,^31^,^32^. Angiogenesis is implicated in the formation of neurovascular units and functional recovery after ischaemic stroke and thus may significantly contribute to a favourable clinical outcome for stroke patients^4^. Our work indicates that 15-LO/15-HETE promotes angiogenesis in response to MCAO by modulating the proliferation of ECs in brain vasculature (Fig. 2). The intensity of ECs in brain vascular increased after MCAO, especially on the 21st day after stroke, along with increased 15-LO expression, and all these effects were reversed by 12/15-LOKO, suggesting that 15-LO could promote angiogenesis in the later stages of ischaemic injury. Our results are consistent with those of a previous study that showed that 15-LO/15-HETE stimulates angiogenesis in ECs and stromovascular tissues derived from adipose tissue^33^.

Previous studies have shown that hypoxia stimulates pulmonary arterial ECs proliferation, which is a key component of vascular angiogenesis^34^. There are reports that arachidonic acid metabolites stimulate growth and migration in vascular ECs^35^. Consistent with this notion, we have found that OGD-enhanced 15-HETE induces BMVEC tube formation and migration *in vitro*. These results are also supported by our cell-cycle studies, in which 15-HETE promoted cell transition from the first gap phase (G_1_ phase) to the DNA replication phase (S phase) and mitotic phase (M phase). The cell-cycle progression from the G_0_/G_1_ phase to S phase is through direct or indirect stimulation of cyclins, including the accumulation of cyclin A and cyclin D^22^. In our study, OGD increased the expression of cyclin A and cyclin D, which was diminished by 15-LO siRNA. Therefore, the effects of OGD may result at least in part from 15-LO-dependent actions. The results suggest that the actions of 15-HETE on the cell-cycle progression are important in OGD-induced proliferation of BMVECs and in the development of angiogenesis, a novel observation worthy of further investigation.

The signalling pathway modulated by 15-HETE in mediating angiogenesis was studied using an inhibitor of PI3K/Akt (LY-294002). PI3K/Akt regulates differentiation of adipocytes^36^. Studies have shown a role of PI3K/Akt in mediating angiogenesis in response to several stimuli, including hypoxia and nitric oxide^37^. The PI3K/Akt signalling pathway regulates ECs survival, migration, and capillary-like structure formation, which are critical steps in angiogenesis^38^. Our results suggest that the PI3K/Akt pathway is modulated by 15-HETE in ECs derived from brain vasculature. This was evidenced by the tube formulation study, BrdU incorporation, PCNA expression and the cell-cycle progression assay. However, whether any of the other downstream effectors of PI3K/Akt are involved in the process is still unknown.

In summary, our data show that the 15-LO expression is apparently increased in later stages of post-stroke mice, 15-LO/15-HETE is involved in the angiogenesis of stroke, and 15-LO/15-HETE provides a microenvironment that activates endogenous restorative mechanisms in the ischaemic brain. Furthermore, our findings suggest that the PI3K/Akt signalling pathway is necessary for the effects of 15-HETE in the regulation of BMVECs angiogenesis. Thus, a novel option focusing on 15-LO/15-HETE-induced angiogenesis is provided to design therapeutic strategies for ischaemic stroke in the future.

## Materials and Methods

For detailed Materials and Methods, please see the Supplemental Data.

### Reagents and animals

Enhanced chemiluminescence (ECL Plus) reagents were obtained from Amersham International (Amersham, UK). Bromodeoxyuridine (BrdU) proliferation assay kit was purchased from Millipore Corporation (Billerica, MA, USA). All other reagents were from common commercial sources.

Genetic control (wild-type, C57BL/6) and 12/15-LO^−/−^ mice on a C57BL/6 background (Strain name: B6.129S2-Alox15tm1Fun; Stock Number: 002778) were obtained from Jackson Laboratories (Bar Harbor, ME, USA), which are fully accredited by the Institutional Animal Care and Use Committee (IACUC). All 12/15-LO^−/−^ mice used in this study were genotyped using DNA isolated from tail biopsy and the following primers: common forward, 5′-GGC TGC CTG AAG AGG TAC AG-3′; wild type reverse, 5′-CCA TAG ACG AGA CCA GCA CA-3′; and mutant reverse, 5′-GGG AGG ATT GGG AAG ACA AT-3′. They were housed 5 per cage on an inverted 12-hour light/dark cycle (light on at 08: 00 p.m.) in an animal facility maintained at ambient temperature of 21 ± 1 °C. Mice were provided with food and water ad libitum. All experimental procedures were carried out in accordance with the guidelines Institutional Animal Care and Use Committees (IACUC) which were approved by Harbin Medical University College of Medicine Institutional Animal Care and Use Committee.

### Ischaemic stroke model (MCAO) and animal experimental groups

The ischaemic stroke model (focal ischaemia) was performed on 9-week-old mice (20–25 g) by transient (60 min) right middle cerebral artery occlusion (MCAO) followed by reperfusion as described previously^39^. An effort was made to minimize the pain and suffering of experimental animals. The regional cerebral blood flow (CBF) was monitored by laser Doppler flowmetry (Perimed, Craponne, France) to control MCAO severity and reperfusion. To perform surgery, mice were rapidly sedated with 4% isoflurane anaesthesia, and the level of sedation was confirmed by the lack of response to tail pinch. Surgery was performed under 1% continuous isoflurane anaesthesia. At the end of ischaemia (60 min MCAO), the animal was briefly re-anesthetized and reperfusion was initiated by filament withdrawal. Animals presenting with sustained CBF reduction >70% during ischaemia or a severe brain haemorrhage after MCAO were excluded from the study (<1%). Sham-operation was performed by inserting the thread into the common carotid artery without advancing it to occlude the MCA. Body temperature was maintained at 37 ± 0.5 °C (confirmed by rectal measurement) and all surgical procedures were performed under sterile conditions. In each experiment, mice were randomly divided into 6 groups: sham-operated group (WT mice), four groups of WT mice which were sacrificed on 1st, 7th, 14th or 21st day after MCAO (n = 10 at each time point), respectively, and the 12/15-LO^−/−^ mice (raised until 21st day after MCAO)^18^,^40^,^41^.

### Cell culture

Brain microvascular endothelial cells (BMVECs) were purchased from ScienCell Research Laboratories (Carlsbad, CA, USA), and maintained in RPMI 1640 medium supplemented with 15% foetal bovine serum (FBS). The identity of the dissociated BMVECs was verified with Factor VIII immunocytochemistry (Sigma-Aldrich). Cells between passages 2 and 4 at 80% confluence were used for experiments. The cells were synchronized in the same cell cycle by serum withdrawal 24 h before each experiment.

### Oxygen-glucose deprivation (OGD) and re-oxygenation

Cell cultures were rinsed twice with glucose-free Hank’s balanced salt solution (HBSS, 5.4 mM KCl, 140 mM NaCl, 2 mM CaCl_2_, 10 mM HEPES, 30 μM glycine, pH 7.4). Cultured BMVECs were incubated in the pre-gassed HBSS buffer and then transferred into an anaerobic chamber which was previously flushed with mixed gas of 5% CO_2_ and 95% N_2_. Cells were maintained in the hypoxic chamber at 37 °C for 60 min. The OGD treatment was stopped by replacing HBSS with 15% FBS. The plate was returned to normoxic conditions and incubated for 24 h for re-oxygenation. Control culture cells were incubated with the HBSS buffer supplemented with 15 mM glucose in a humidified incubator at normoxia for the same period as the OGD cultures.

### Statistical analysis

The data are presented as the mean ± standard error of mean (SEM). Statistical analysis was performed with Student’s *t*-test or one-way ANOVA followed by Dunnett’s test where appropriate. *p* < 0.05 was considered statistically significant.

## Additional Information

**How to cite this article**: Wang, D. *et al*. Key role of 15-LO/15-HETE in angiogenesis and functional recovery in later stages of post-stroke mice. *Sci. Rep.* **7**, 46698; doi: 10.1038/srep46698 (2017).

**Publisher's note:** Springer Nature remains neutral with regard to jurisdictional claims in published maps and institutional affiliations.

## Supplementary Material

Supplementary Information

## Figures and Tables

**Table 1:** Neurological deficit scores.

	Score					Average score
	0	1	2	3	4	
Sham control	10	—	—	—	—	0
MCAO 1d	—	1	4	5	—	2.4 ± 0.69*
MCAO 7d	—	3	4	3	—	2 ± 0.28*
MCAO 14d	—	4	5	1	—	1.7 ± 0.67*
MCAO 21d	—	7	3	—	—	1.3 ± 0.48*
MCAO 21d+12/15-LO^−/−^	—	4	4	2	—	1.8 ± 0.79*

Abbreviation: MCAO = middle cerebral artery occlusion.
