# Supplementary material for: Key role of 15-LO/15-HETE in angiogenesis and functional recovery in later stages of post-stroke mice
Source: Sci Rep. 2017 Apr 24;7:46698. doi: 10.1038/srep46698 (PMC5402258; doi:10.1038/srep46698)

Key role of 15-LO/15-HETE in angiogenesis and functional recovery in later stages of post-stroke mice

Authors:

Di Wang[1], Yu Liu[1], Li Chen[1], Pengyan Li[1], Youyang Qu[1], Yanmei Zhu[1], *Yulan Zhu[1]

[1]The Second Affiliated Hospital of Harbin Medical University, Department of Neurology

Corresponding Author:

Yulan Zhu

Department of Neurology, The Second Affiliated Hospital of Harbin Medical University

246 Xuefu Road, Harbin, Heilongjiang 150086, China

Tel: 86-451-86297470, 86-18645049917

Fax: 86-451-86605656

E-mail: ylz0451@gmail.com

Search Terms: 15-lipoxygenase, 15-hydroxyeicosatetrienoic acid, cerebral ischemia/reperfusion injury, angiogenesis, PI3K/Akt

Author Contributions:

Di Wang, study concept and design, acquisition of data, analysis and

interpretation of data

Yu Liu, study concept and design, acquisition of data

Li Chen, acquisition of data

Pengyan Li, acquisition of data

Youyang Qu, critical revision of manuscript for intellectual content

Yanmei Zhu, critical revision of manuscript for intellectual content

Yulan Zhu, study concept and design, critical revision of manuscript for Intellectual content

Author Disclosures:

All authors report no disclosures

Supplemental data

Neurological deficit scores

Neurological deficit scores (NDS) were performed on 1st, 7th, 14th or 21st day after MCAO right before decapitation. Bederson scores were used to evaluate global neurological function as previously described[1]. The scoring system was as follows: 0, no deficit; 1, forelimb weakness and torso turning to the ipsilateral side when held by tail; 2, circling to affected side; 3, unable to bear weight on affected side; and 4, no spontaneous locomotor activity or barrel rolling.

Infarction volume measurement

The infarct analysis cohort was subjected to stroke, mice were euthanized by decapitation at different time point after reperfusion. Briefly, brains were removed and cut into five 2-mm coronal sections and stained with 1.5% 2, 3, 5-triphenyltetrazolium chloride (TTC) for 8 min at 38 °C. Slices were formalin-fixed (4%), digitalized and infarct volumes were analysed (Sigma Scan Pro) as previously described[2]. The final infarct volumes are presented as a percentage (percentage of contralateral structures with correction for edema) ± SD, as previously described (n = 10/group)[2,3].

Adhesive-tape removal test

The adhesive-tape (sticky-tape) removal test is a measure of somatosensory dysfunction after cerebral ischaemia in mice[4]. Adhesive-backed tape (30 x 40 mm) was used as tactile stimuli placed on the distal-radial region of the left wrist, and the mean time to remove the tape was recorded. Animals were trained for 5 days once a day prior to stroke, and the latency to remove adhesive tape was measured on days 0, 1, 7, 14 and 21 after stroke (n = 10/group).

Accelerated rotarod test

The rotarod test is used to assess motor coordination and balance alterations after ischaemic brain injury in the rodent[5]. The rotarod apparatus consists of a striated rod (diameter 3 cm) subdivided into 5 areas (width 5 cm) by disks 25 cm in diameter. Mice (n = 10/group) were conditioned to the accelerating rotarod (Ugo Basile, France) for 5 days before MCA occlusion. To this end,

mice were first placed on the apparatus during 30 s with no rotation and thereafter for 2 min with a constant low speed (4 rpm). They were tested until they achieved a criterion of remaining on the rotating spindle for 1 min. This procedure was performed only the first day of training. After 10 min rest, each mouse then received a single baseline trial on the accelerating rotarod in which the spindle increased in speed from 4 to 40 rpm over a period of 6 min. The test trial was performed at different times after MCAO in accordance with adhesive-tape removal test. The maximum duration the animals were able to walk on the rotarod before falling was measured (maximum value 6 min). Mice were tested over 3 daily trials in the accelerated condition (4-40 rpm). The daily mean value was taken for each mouse and used for statistical analysis.

Immunohistochemistry

Mice were euthanized by decapitation at different time point after reperfusion. Then we removed the brains and immersed them in 4% paraformaldehyde for overnight fixation. Then the tissues were dehydrated, cleared and embedded in paraffin wax. The paraffin blocks were cut into 5 μm thick sections. For immunohistochemistry, sections were deparaffinized and rehydrated in graduated alcohol. Then they were placed in sodium citrate buffer (0.1 mol/L, pH 6.0) and heated for 2 min for antigen retrieval, and then the sections were incubated with anti-15-LO (1:100) antibodies. After overnight incubation, the sections were exposed to the secondary antibodies (1:200) for the IgGs. Sections were visualized with 3, 3-diaminobenzidine and counterstained using

haematoxylin. Brown and yellow colours indicated positive stains.

Measurement of 15-HETE level

To examine whether OGD promotes the generation of endogenous 15-HETE in BMVECs, the15(S)-HETE EIA Kit (Catalog No.534721, Cayman) was performed for the detection of the amount of 15(S)-HETE. Cell pellets were lysed and supernatants concentrations were determined by Bradford protein assay. The results were analyzed by Cayman Chemical Company Enzyme Immunoassay (EIA) Tools.

Double immunofluorescence staining

After dissecting out brains from mice, the brain tissues were fixed in 4% paraformaldehyde for 6 h, transferred to 10%, 20%, 30% sucrose in 0.1 mol/L phosphate buffer (pH 7.4) for 12 h respectively in order to cryoprotection, and stored at 4°C. Brain tissue was frozen in Tissue-Tek OCT compound (Sakura Finetechnical Co) at -20°C. Then, Cryosections (5 μm) were made using Leica Kryostat (Model: CM3050S, Leica). The cryosections were blocked with 10% normal goat serum/PBS for 30 min. CD31 and Ki67 was incubated at 4°C overnight. After washing three times with PBS, sections were incubated with Alexa Fluor 488 conjugated mouse anti-goat antibody (Invitrogen, 1: 100) and Alexa Fluor 546 conjugated goat anti-rabbit antibody (Invitrogen, 1: 100) for 2 h at room temperature and DAPI away from light. Sections were washed three times with PBS and then examined with a microscope (Olympus, Japan), and images were recorded by digital photomicrography (Olympus, Japan).

SiRNA design and transfection

To block the expression of 15-LO protein, BMVECs were transfected with the corresponding siRNAs, which were designed and synthesized by GenePharma using X-tremeGene siRNA Transfection Reagent (Roche Applied Science, Mannheim, Germany). Non-targeted control siRNA (siNC) was used as the negative control. The sense sequence of siRNA against 15-LO and siNC sequence was listed below: accession no. ds-siRNA sequence corresponding nucleotides, 15-LO: (NM_001034472.3) 5'-GCUUUGAUUGGUGAUGUUATT-3', siNC: 5'-UUCUGAGAACGUGACACGUTT-3'. Cells were cultured till 30-50% confluence, 1.5 μg siRNA and 7.5 μL X-tremeGene siRNA Transfection Reagent were diluted in the serum-free Opti-MEM-1 medium, and after 5 min, we mixed them together. Next, we incubated the mixture (siRNA/transfection reagent) for 20 min then added it directly to the cells. After 4-6 h exposure to siRNA, the transfection reagents were removed and cell culture continued in DMEM containing 5% FBS for another 24 h under normoxic (21% O_2/5% CO_2/balance N_2) or OGD growth conditions as required.

Immunocytochemistry

BMVECs were cultured on coverslips, which were covered in 24-well culture plates with polylysine. After treatment, cells were fixed with 4% paraformaldehyde, permeabilized with 0.5% Triton X-100 for 10 min, blocked with 3% normal bovine serum at 37°C for 30 min, and incubated with

anti-15-LO primary antibody (rabbit, 1:50) and anti-CD31 primary antibody (rat, 1:100) at 4°C overnight. After washing three times with PBS, the cells were incubated with FITC-conjugated secondary antibody (1:100) for 2 h and DAPI for 10 min. in the dark. The images were recorded by digital photomicrography (Olympus, Japan).

Tube formulation assay

96-well culture plates (Costar, Corning) were coated with growth factor-reduced Matrigel (BD Biosciences) in a total volume of 30μl and allowed to solidify for 30 min at 37°C. BMVECs were trypsnized and resuspended at 5 × 10^4 cells/ml and 200 μl of this cell suspension were added into each well. Tube formation was observed under an inverted microscope (Nikon, Japan). Tube length was measured using the Image-Pro Plus 6.0 (Media Cybernetics, USA).

Bromodeoxyuridine incorporation

BMVECs were plated in 96-well plates at the density of 1 × 10^4 cells/well, and then subjected to growth arrest for 24 h before being exposed to OGD or treated with different agents in 5% FBS-DMEM. We measured Brdu incorporation according to the Millipore Brdu proliferation assay kit instruction. Briefly, the cells were labeled with 10 ng/ml of Brdu during incubation, washed 3 times with cold wash buffer, fixed, air-dried and incubated for 1 h at room temperature with mouse anti-Brdu monoclonal antibody (diluted 1:200). The antibody was aspirated. The cells were washed 3 times and then incubated

with peroxidase goat anti-mouse IgG (1:2000) at room temperature for 30 min. The cells were washed 3 times, and 100 μl substrates were added to each well and incubated for 30 min in darkness. Thereafter, the absorbance of samples was recorded at dual-wave lengths of 450 to 540 nm.

Western blotting

Proteins were extracted from BMVECs, using the procedures essentially the same as described in detail elsewhere. Protein samples (30–50 μg) were fractionated by SDS-PAGE (8-10% polyacrylamide gels) using primary antibodies against 15-LO, PCNA, cyclin A, cyclin D, and PI3K, with β-actin as an internal control. Immunoblots were quantified with Quantity One software (Bio-Rad Laboratories, Hercules, CA, USA).

Cell cycle analysis

The Cycle TEST™ PLUS DNA Reagent Kit was used for examining whether the cell cycle was influenced by OGD or 15-HETE. After the cells were subjected to drugs or OGD, they were harvested by trypsinization, then centrifuged and suspended with 1 ml of cold PBS. Following two washes with PBS, the cells were resuspended and fixed using 70% ethanol. The ethanol was removed and the cells were incubated in 200 μl PBS. The cells were stained with propidium iodide according to the kit protocol. DNA fluorescence was measured, and flow cytometry proceeded using a BD FACSCalibur flow cytometer (Bedford, MA, USA). For each sample, 2×10^4 events were accumulated in a histogram. The proportions of cells in the different phases of

the cell cycle were calculated from each histogram.

Supplemental References

1. Chen, L. et al. Tongxinluo attenuates neuronal loss and enhances neurogenesis and angiogenesis in the ipsilateral thalamus and improves neurological outcome after focal cortical infarction in hypertensive rats. Restor Neurol Neurosci 2014;32:533-546.
2. Venna, V.R. et al. NF-kappaB contributes to the detrimental effects of social isolation after experimental stroke. Acta Neuropathol 2012b;124:425-438.
3. Li, J., Benashski, S.E., Venna, V.R. & McCullough, L.D. Effects of metformin in experimental stroke. Stroke 2010;41:2645-2652.
4. Bouet, V. et al. The adhesive removal test: a sensitive method to assess sensorimotor deﬁcits in mice. Nat Protoc 2009;4:1560-1564.
5. Rogers, D.C., Campbell, C.A., Stretton, J.L. & Mackay, K.B. Correlation between motor impairment and infarct volume after permanent and transient middle cerebral artery occlusion in the rat. Stroke 1997;28:2060-2065.

Supplementary Datasets

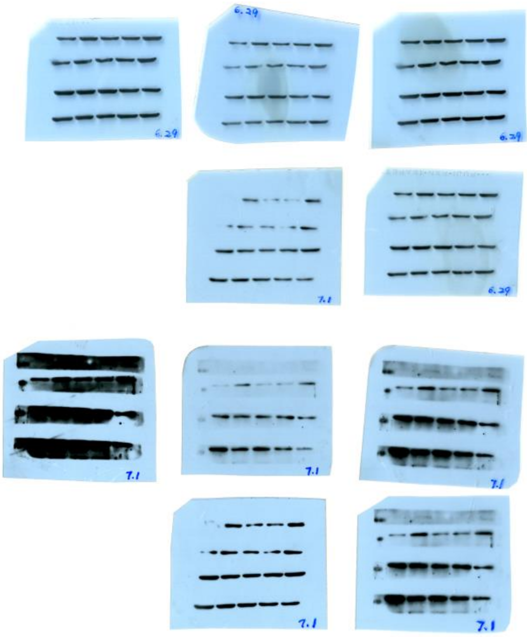
6.29
6.29
6.29
6.29
7.1
6.29
7.1
7.1
7.1
7.1
7.1

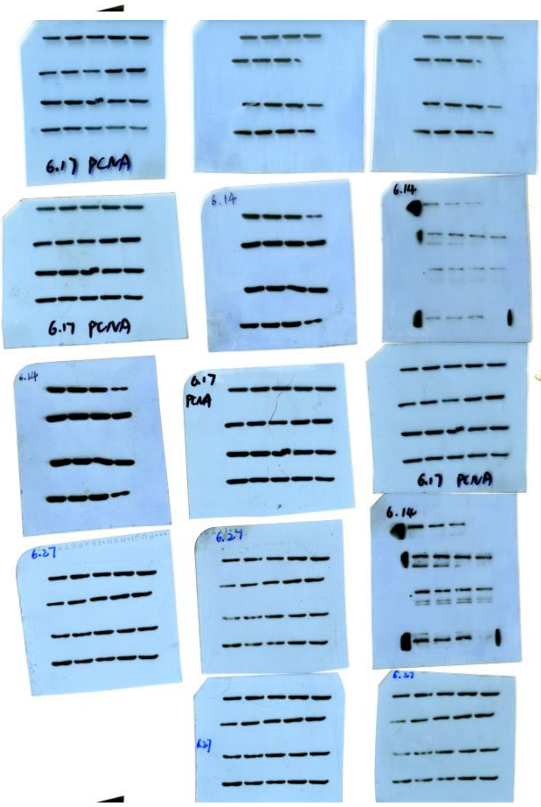
6.17 PCNA
6.17 PCNA
6.14
6.14
6.14
6.17 PCNA
6.17 PCNA
6.14
6.27
6.27
6.27

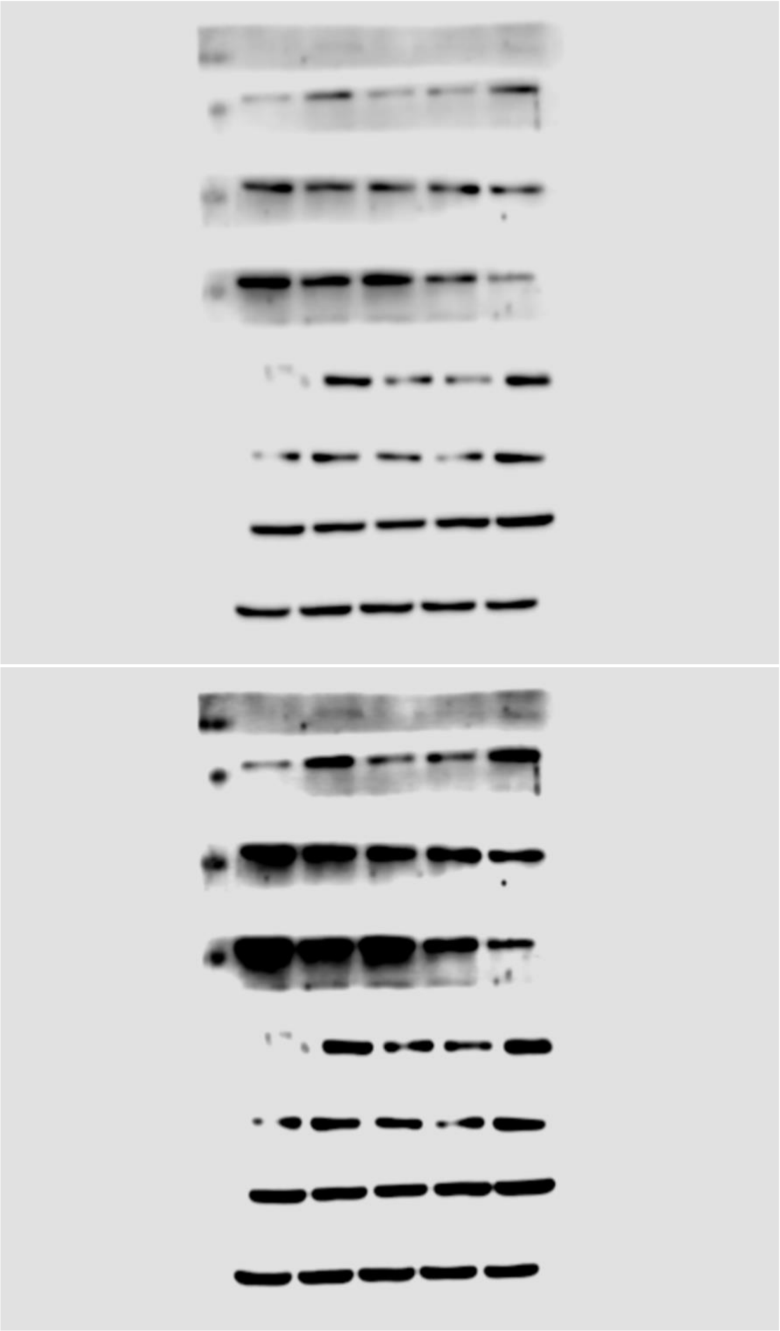

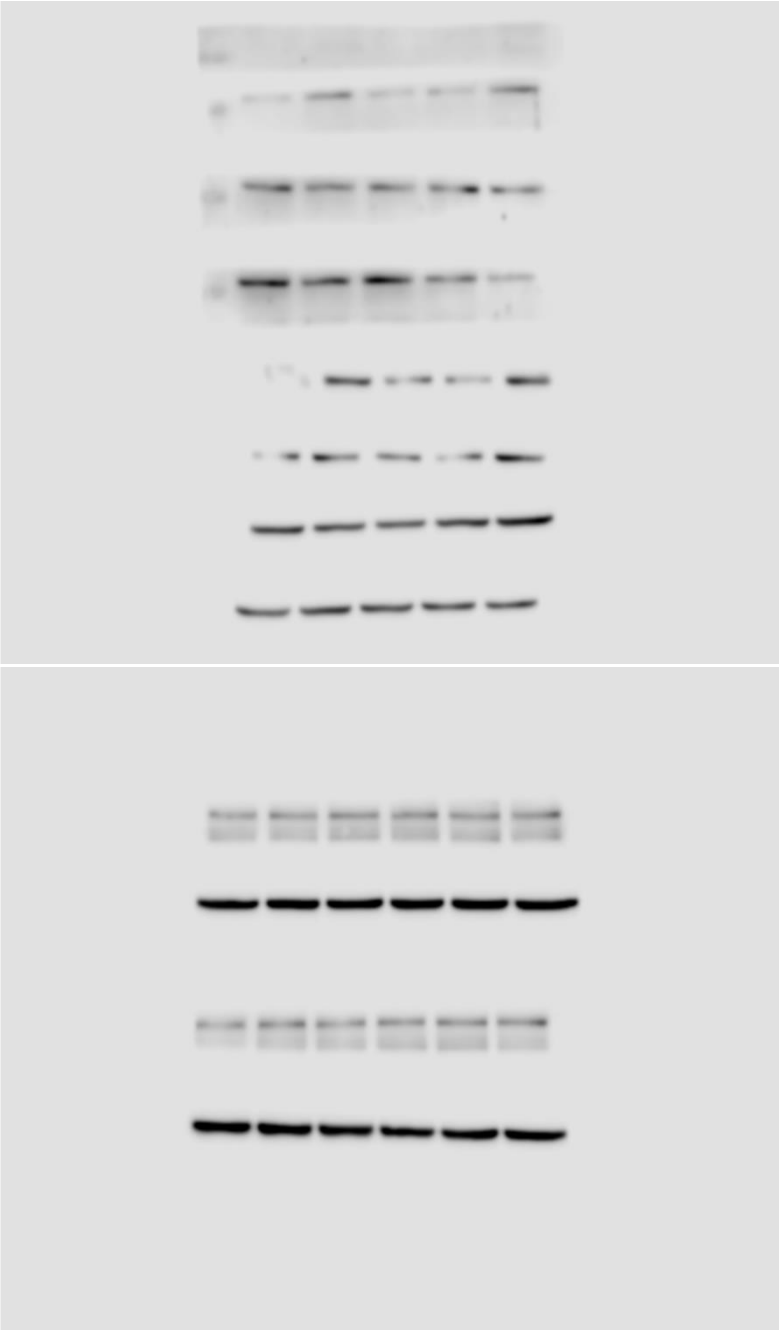

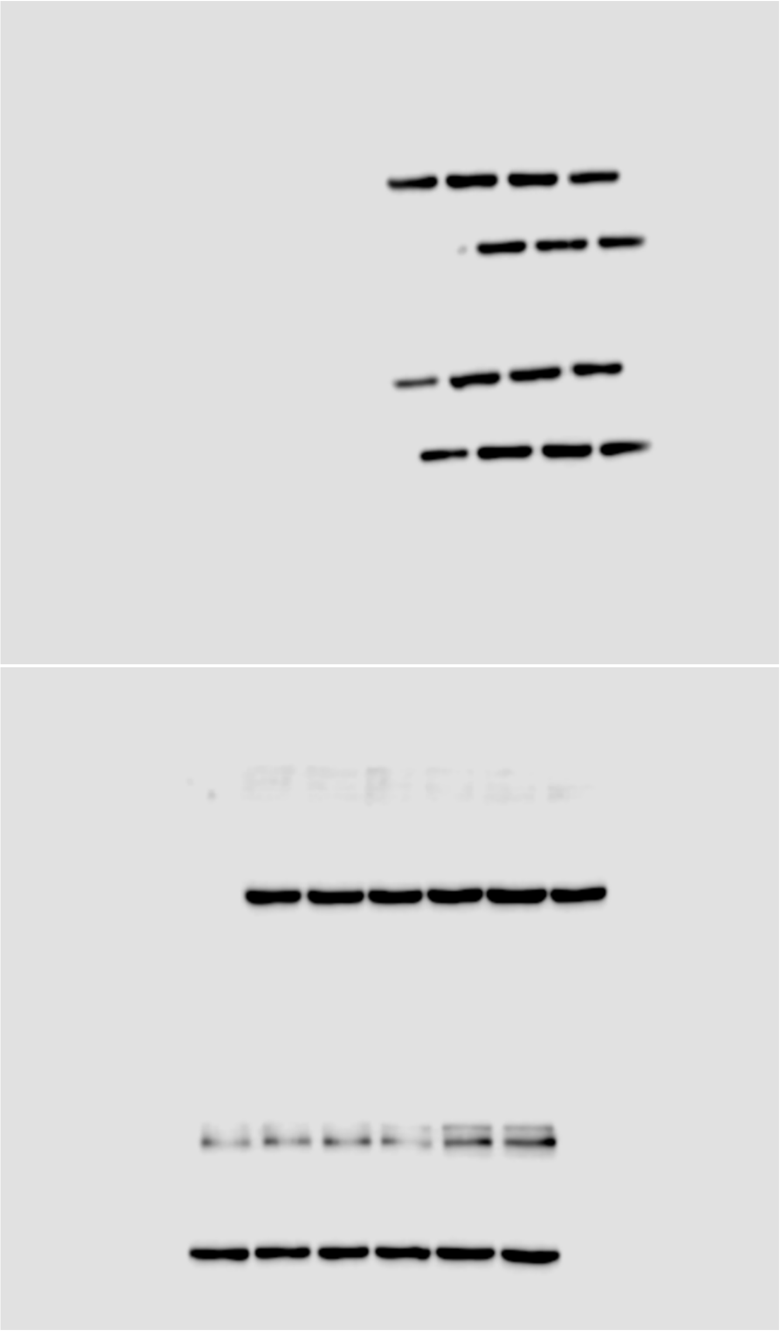

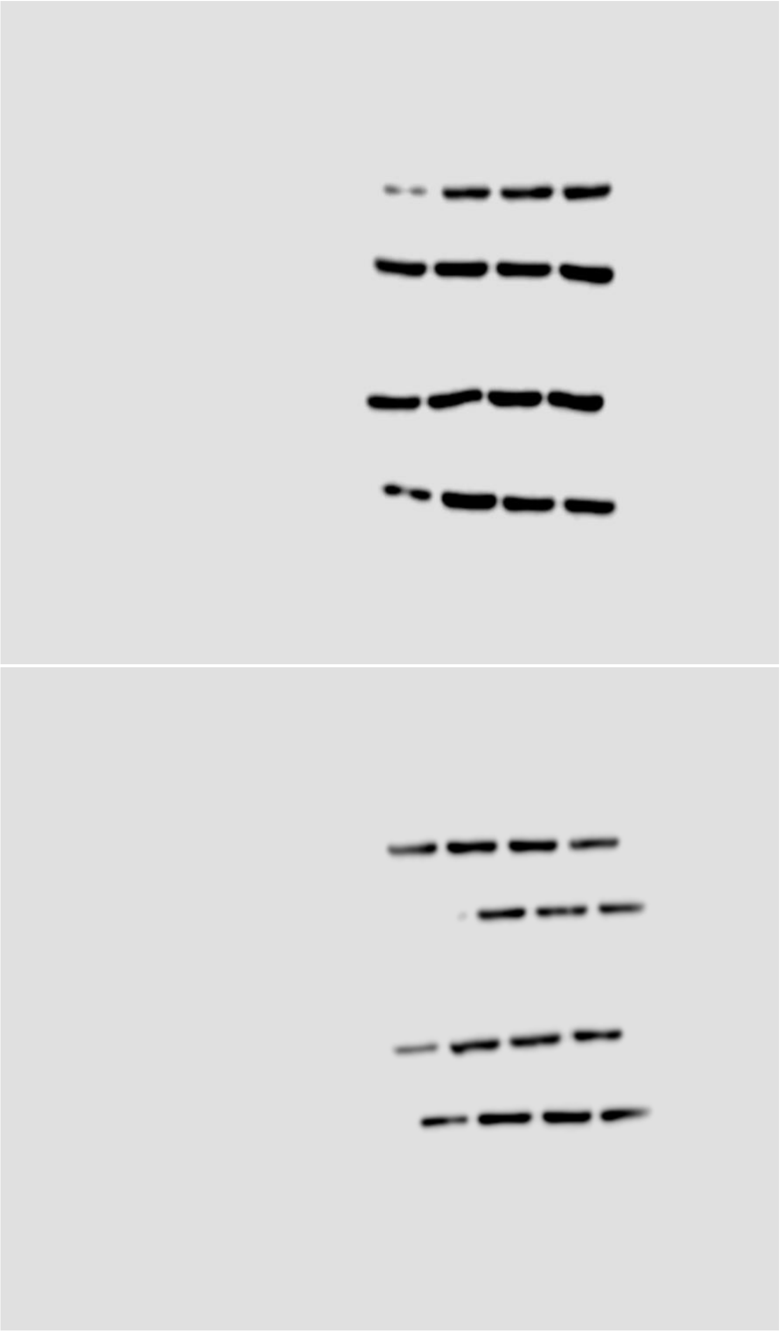

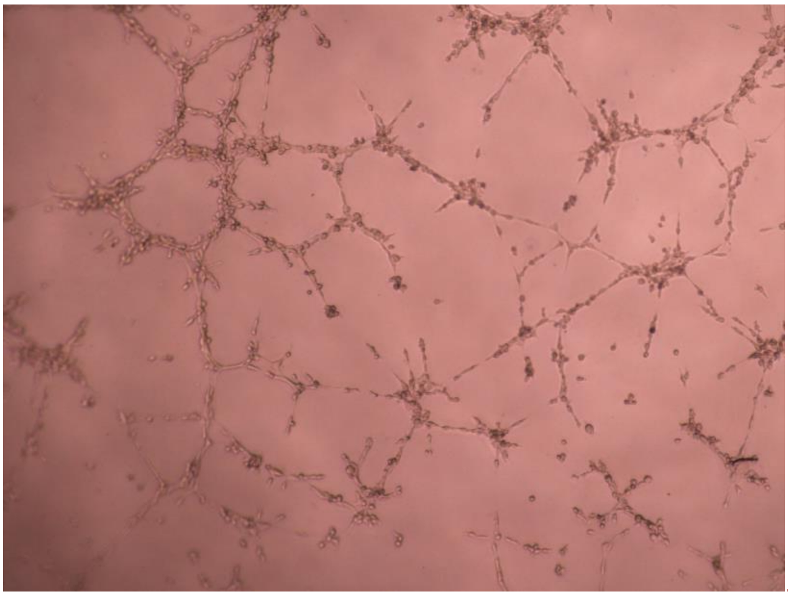
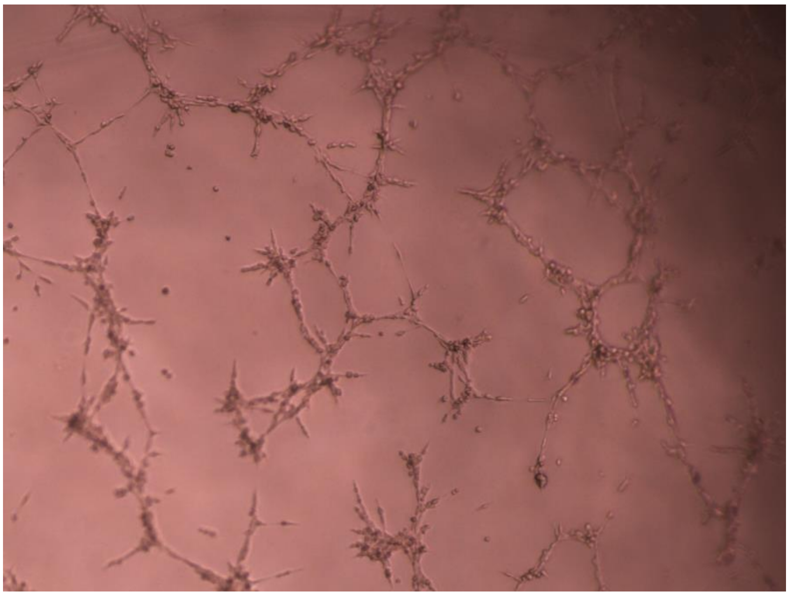
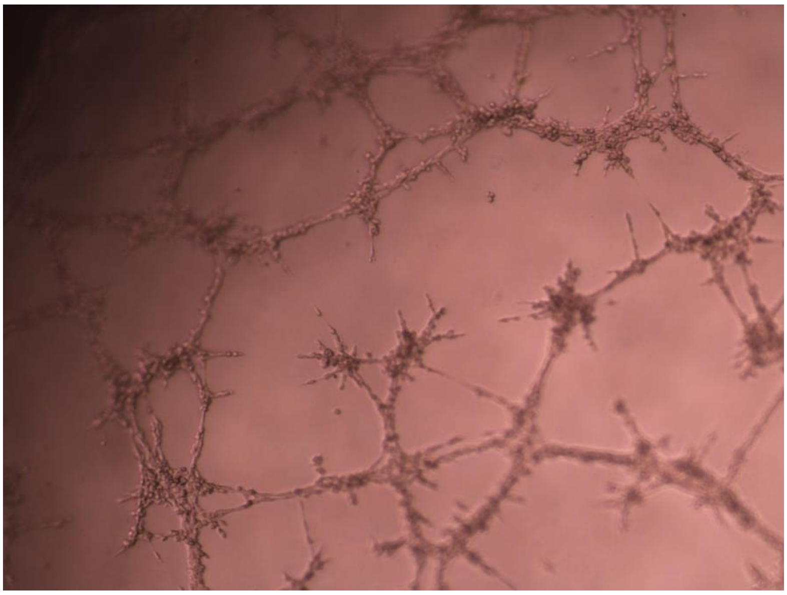

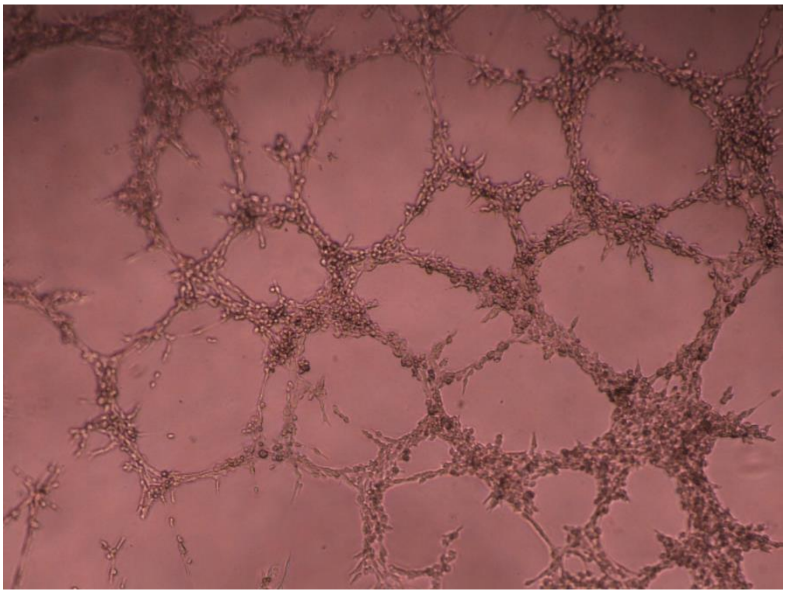

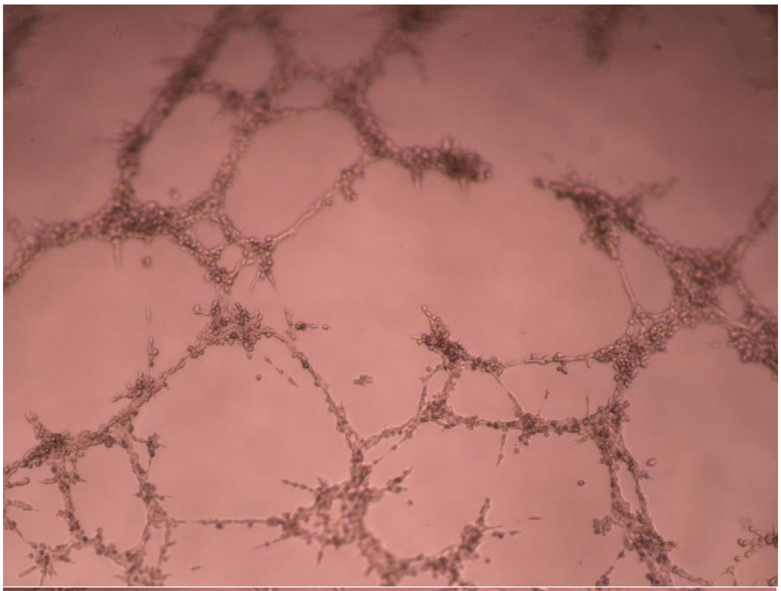

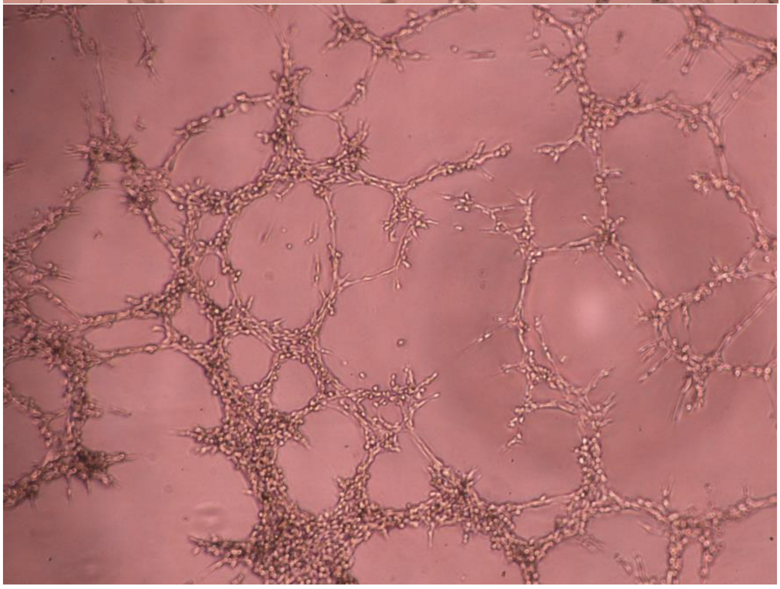

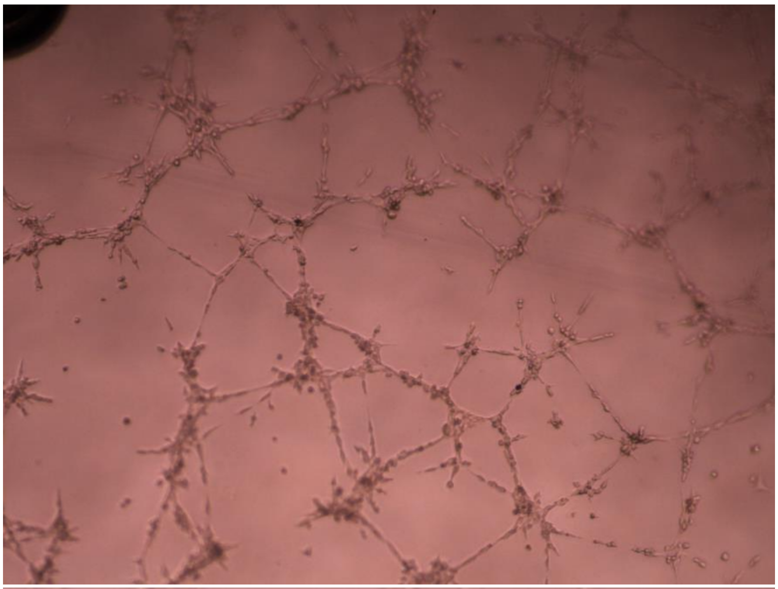
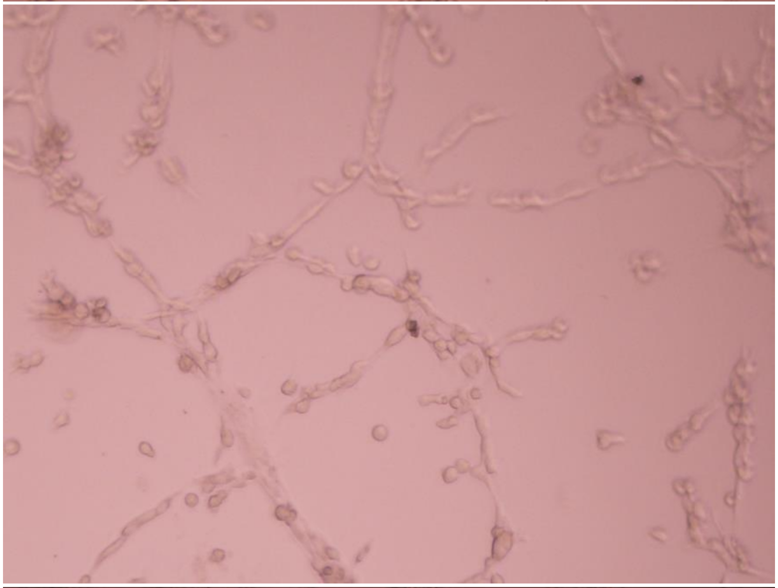
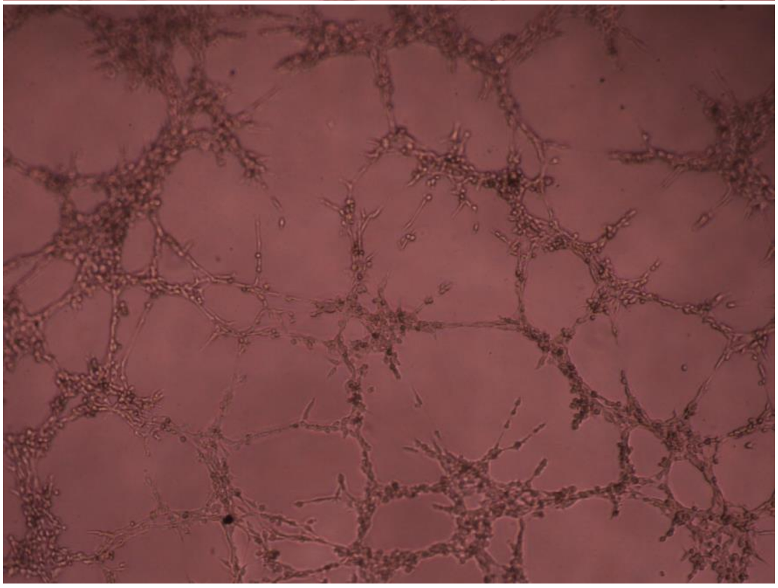

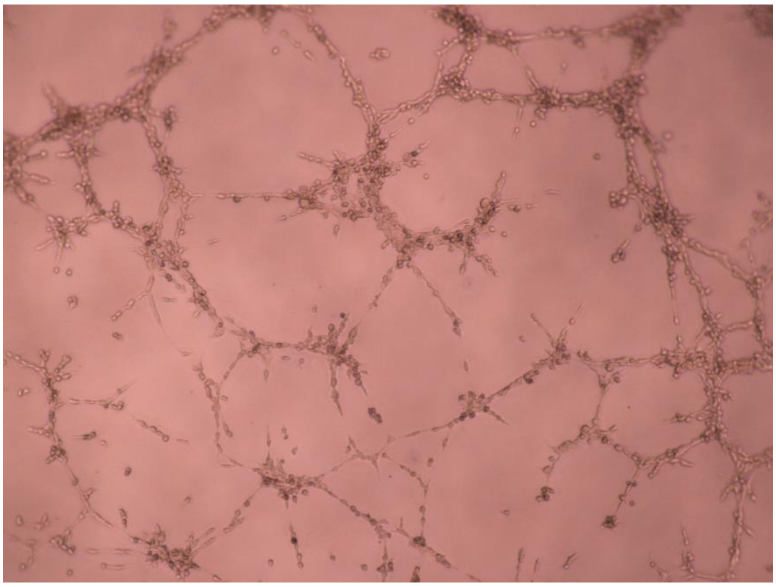

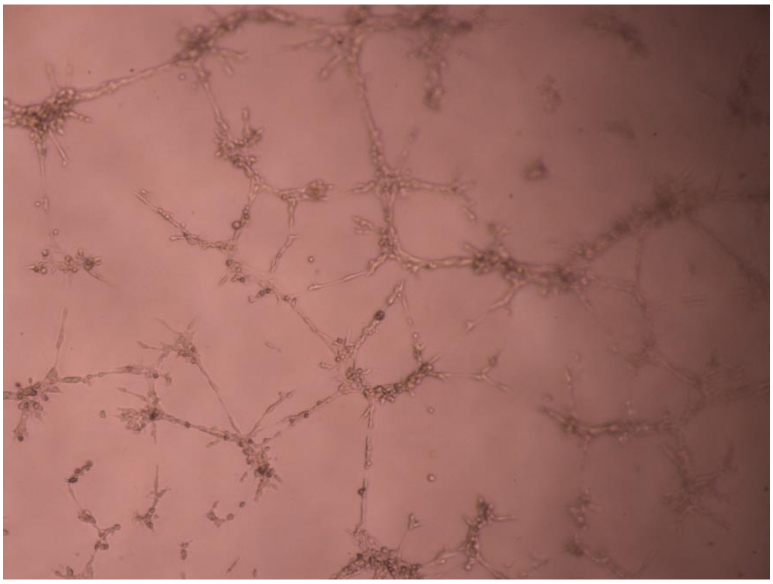

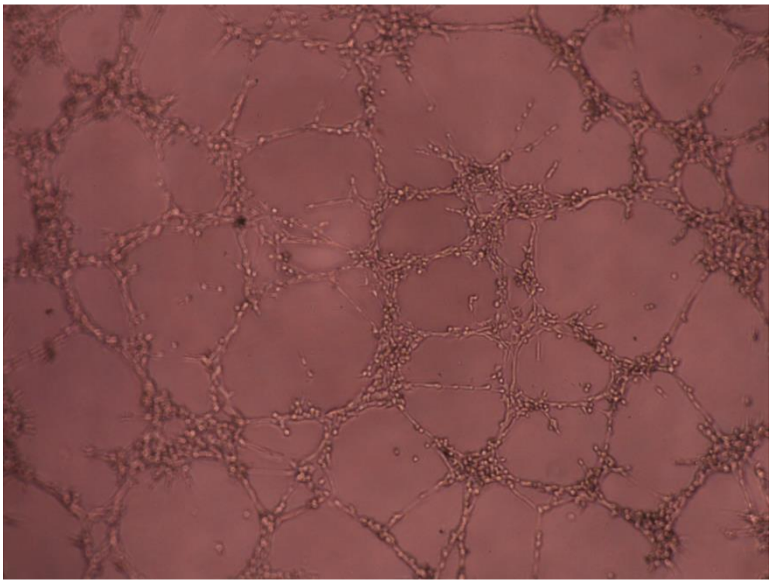

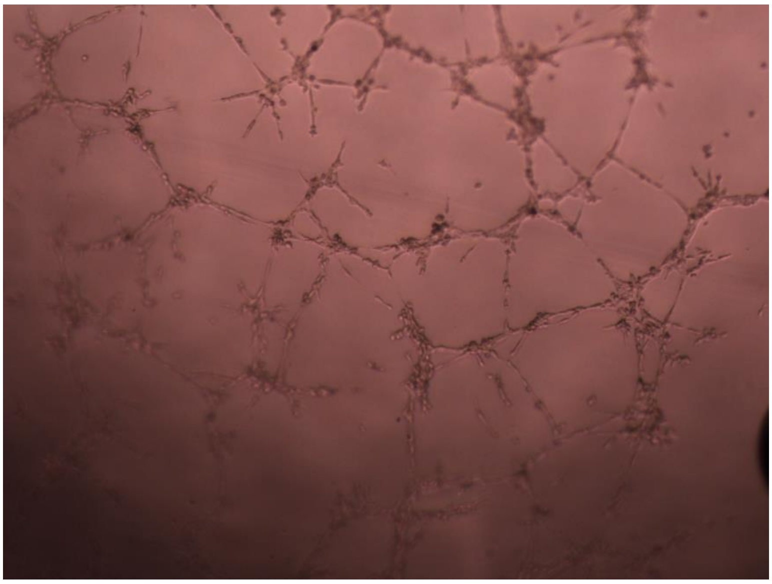

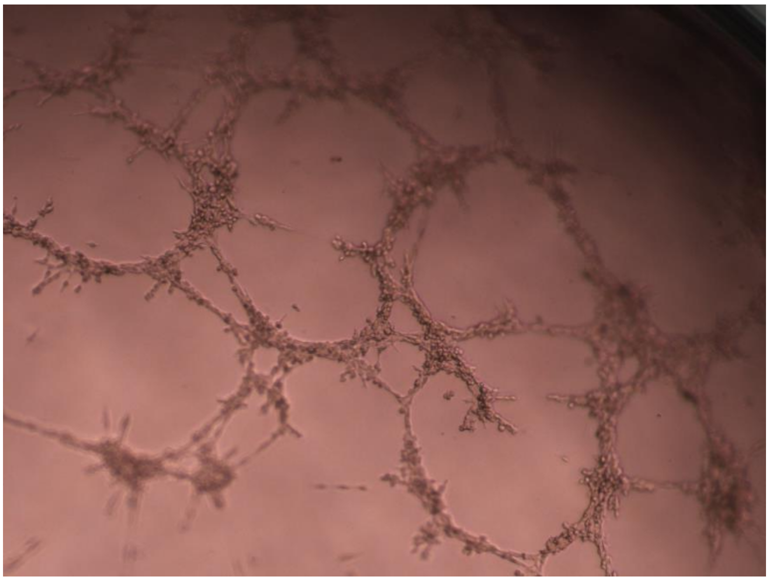

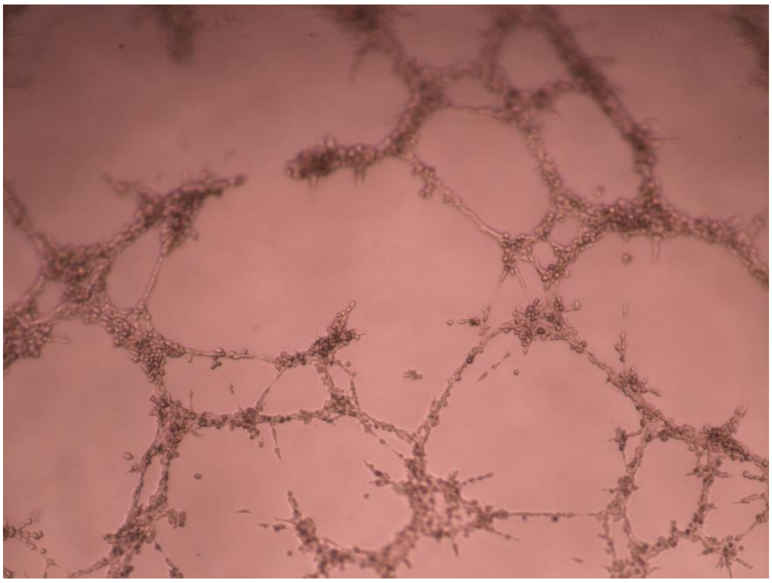

Supplement: Supplementary Information [file srep46698-s1.pdf]
